# Utility of Post-Mortem Genetic Testing in Cases of Sudden Arrhythmic Death Syndrome

**DOI:** 10.1016/j.jacc.2017.02.046

**Published:** 2017-05-02

**Authors:** Najim Lahrouchi, Hariharan Raju, Elisabeth M. Lodder, Efstathios Papatheodorou, James S. Ware, Michael Papadakis, Rafik Tadros, Della Cole, Jonathan R. Skinner, Jackie Crawford, Donald R. Love, Chee J. Pua, Bee Y. Soh, Jaydutt D. Bhalshankar, Risha Govind, Jacob Tfelt-Hansen, Bo G. Winkel, Christian van der Werf, Yanushi D. Wijeyeratne, Greg Mellor, Jan Till, Marta C. Cohen, Maria Tome-Esteban, Sanjay Sharma, Arthur A.M. Wilde, Stuart A. Cook, Connie R. Bezzina, Mary N. Sheppard, Elijah R. Behr

**Affiliations:** a Heart Centre, Department of Clinical and Experimental Cardiology, Academic Medical Center, Amsterdam, the Netherlands; b Molecular and Clinical Sciences Research Institute, St. George's, University of London, London, United Kingdom; c Cardiology Clinical Academic Group, St. George’s University Hospitals NHS Foundation Trust, London, United Kingdom; d National Heart and Lung Institute, Sydney Street, Imperial College London, London, United Kingdom; e Royal Brompton & Harefield Hospitals NHS Foundation Trust, London, United Kingdom; f Cardiovascular Genetics Center, Department of Medicine, Montreal Heart Institute and Université de Montréal, Montreal, Canada; g Cardiac Inherited Disease Group New Zealand, Green Lane Paediatric and Congenital Cardiac Services, Starship Children's Hospital, Auckland New Zealand; The University of Auckland, Department of Paediatrics Child and Youth Health, Auckland, New Zealand; h National Heart Centre Singapore, Singapore; i Department of Cardiology, Rigshospitalet, Copenhagen, Denmark; j Sheffield Children's NHS Foundation Trust, Sheffield, United Kingdom; k Princess Al-Jawhara Al-Brahim Centre of Excellence in Research of Hereditary Disorders, Jeddah, Kingdom of Saudi Arabia; l Duke-National University of Singapore, Singapore

**Keywords:** cardiomyopathy, channelopathy, molecular autopsy, next-generation sequencing, unexplained sudden death, ACM, arrhythmogenic cardiomyopathy, ACMG, American College of Medical Genetics, BrS, Brugada syndrome, CPVT, catecholaminergic polymorphic ventricular tachycardia, LQTS, long QT syndrome, MAF, minor allele frequency, SADS, sudden arrhythmic death syndrome, SCD, sudden cardiac death, VUS, variant of unknown significance

## Abstract

**Background:**

Sudden arrhythmic death syndrome (SADS) describes a sudden death with negative autopsy and toxicological analysis. Cardiac genetic disease is a likely etiology.

**Objectives:**

This study investigated the clinical utility and combined yield of post-mortem genetic testing (molecular autopsy) in cases of SADS and comprehensive clinical evaluation of surviving relatives.

**Methods:**

We evaluated 302 expertly validated SADS cases with suitable DNA (median age: 24 years; 65% males) who underwent next-generation sequencing using an extended panel of 77 primary electrical disorder and cardiomyopathy genes. Pathogenic and likely pathogenic variants were classified using American College of Medical Genetics (ACMG) consensus guidelines. The yield of combined molecular autopsy and clinical evaluation in 82 surviving families was evaluated. A gene-level rare variant association analysis was conducted in SADS cases versus controls.

**Results:**

A clinically actionable pathogenic or likely pathogenic variant was identified in 40 of 302 cases (13%). The main etiologies established were catecholaminergic polymorphic ventricular tachycardia and long QT syndrome (17 [6%] and 11 [4%], respectively). Gene-based rare variants association analysis showed enrichment of rare predicted deleterious variants in *RYR2* (p = 5 × 10^-5^). Combining molecular autopsy with clinical evaluation in surviving families increased diagnostic yield from 26% to 39%.

**Conclusions:**

Molecular autopsy for electrical disorder and cardiomyopathy genes, using ACMG guidelines for variant classification, identified a modest but realistic yield in SADS. Our data highlighted the predominant role of catecholaminergic polymorphic ventricular tachycardia and long QT syndrome, especially the *RYR2* gene, as well as the minimal yield from other genes. Furthermore, we showed the enhanced utility of combined clinical and genetic evaluation.

Sudden cardiac death (SCD) in the young is a devastating event. The annual incidence in the 1- to 35-year-old age group is estimated at 1.3 to 2.8 per 100,000 (1). Autopsies in younger SCD victims lead to a diagnosis of structural cardiac disease in the majority. Yet, in 30% to 40% of cases, the cause remains elusive despite toxicological and histopathologic analysis 1, 2. A proportion is expected to have suffered arrhythmic death and is referred to as succumbing from sudden arrhythmic death syndrome (SADS) 3, 4. SADS is caused, in part, by primary electrical disorders such as long QT syndrome (LQTS), Brugada syndrome (BrS), and catecholaminergic polymorphic ventricular tachycardia (CPVT), which are associated with a structurally normal heart 5, 6.

Post-mortem testing of the genes underlying primary electrical disorders in cases of SADS (the “molecular autopsy”) allows for ascertainment of the genetic cause 7, 8. This may inform clinical and genetic evaluation of surviving relatives for SCD prevention (9). Early case series focused on 4 main genes (*KCNQ1*, *KCNH2*, *SCN5A*, and *RYR2*). Relatively small studies have interrogated extended gene panels including cardiomyopathy-associated genes by next-generation sequencing. The yield varied widely due to variation in variant-calling 2, 8, 10. More recently, Bagnall et al. (1) performed genetic testing in 113 unexplained SCDs (i.e., SADS) and the authors reported a “clinically relevant” cardiac genetic variant in 27%. A definite clinical diagnosis (predominantly LQTS and CPVT) was established in 12 of 91 families (13%) who underwent clinical screening.

Critically, however, recent large-scale genetic studies have identified widespread low-frequency genetic variation in cardiac genes that may confound results (11). This necessitates robust filtering strategies in the analysis of candidate variants. Furthermore, there has been limited evaluation of the combined utility of molecular autopsy in clinical and genetic evaluation of the family.

We here investigated an extended panel of 77 primary electrical disorder and cardiomyopathy genes in the largest set of SADS cases (n = 302) thus far. By applying stringent American College of Medical Genetics (ACMG) consensus guidelines for variant classification (12), we identified clinically actionable genetic variants and deduced a likely realistic contribution of the tested genes to SADS. We then evaluated the role of combined post-mortem genetic testing and clinical evaluation in surviving relatives.

## Methods

SADS was defined as an unexplained death without prior cardiovascular disease within 1 h of symptom onset or an unwitnessed death with the individual being seen in good health within 24 h of death; no cause of death was identifiable on comprehensive coronial and cardiac autopsy or on toxicological analyses (13). Cases with structural disease (e.g., cardiomyopathy; n = 53) or nonspecific changes (e.g., left ventricular hypertrophy without disarray or idiopathic fibrosis; n = 29) at autopsy were excluded from the study. Data including clinical history, prior symptoms, circumstance of death, and family history were collected by direct contact with next of kin, and from coroner and pathologist reports. In total, 302 of 384 potential cases (79%) with suitable deoxyribonucleic acid (DNA) were included. Cohort 1 consisted of 2 population-based coronial series: Cardiac Inherited Disease Registry, Auckland, New Zealand, 2000 to 2009 (n = 38) 14, 15; and the SCD Registry, Denmark, 2000 to 2006, (n = 27) (16). Cohort 1 was also drawn from: consecutive referrals for autopsy (Royal Brompton Hospital, United Kingdom 2007 to 2011; n = 28); Sheffield Children’s Hospital (United Kingdom 1985 to 2001; n = 20) and consecutive referrals for familial cardiac evaluation (St. George’s and Lewisham Hospitals, United Kingdom, 2009 to 2011; n = 30); and Academic Medical Centre, Netherlands 1995 to 2011 (n = 10). Cohort 2 consisted of 149 consecutive referrals for autopsy at St. George’s Hospital, United Kingdom, 2012 to 2015. The study was approved by Research Ethics Service Wandsworth.

The control cohort was derived from 1,158 adult volunteers recruited prospectively as part of the UK Digital Heart Project. Cardiac magnetic resonance imaging and an electrocardiogram (ECG) were normal in all subjects and there was no family history of cardiac disease. The study was approved by the National Health Service of England Research Ethics Committee.

### Next-generation sequencing

Subjects’ DNA samples were sequenced using targeted high-throughput sequencing. Target regions were captured by in-solution hybridization target capture, using the SureSelect system (Aligent Technologies, Santa Clara, California) for cohort 1 or the Illumina TruSight Cardio system (Illumina, San Diego, California) for cohort 2 and controls. For SureSelect capture, custom hybridization-capture probes were designed using the eArray platform (Aligent Technologies) to target 201 genes implicated in cardiovascular disease. Ribonucleic acid baits targeted all exons of all Ensembl (v54) (17) transcripts, including untranslated regions, with a 100-bp extension into adjacent introns. The TruSight Cardio Kit consists of comprehensive coverage of 174 genes (coding sequence region only) with known associations to 17 inherited cardiac conditions (18). Libraries were prepared per manufacturer’s instructions and sequenced on the Illumina HiSeq (cohort 1) or NextSeq (cohort 2 and controls). We analyzed 77 genes tested by both systems that have been previously associated with primary electrical diseases or cardiomyopathies (Online Table 1).

All the samples (i.e., cohorts 1, 2, and controls) were processed together. Low quality (Q <20, window size 5) reads/bases were trimmed and read quality assessed. High-quality reads were mapped to UCSC GRCh37/hg19 reference genome. Next-generation sequencing was used to mark duplicate reads, realign locally around indels, and recalibrate base quality scores according to best practices. Alignment summary metrics and coverage and callability metrics were generated. A base was considered “callable” if sequenced with minimum read depth = 10x, base quality ≥20, and mapping quality ≥10. Target base callability of >90% was achieved in 98% of samples overall and in 92%, 95%, and 100% of samples from cohort 1, 2, and controls, respectively. Gene level callability is shown in Online Table 2. Exons with callability <80% are shown in Online Table 3. Genome Analysis Toolkit v3.2 HaplotypeCaller was used to call variants from reads mapped with quality ≥8, and for joint variant calling. Indels with quality by depth <2.0, ReadPosRankSum <−20.0, FisherStrand >200.0 and coefficient of inbreeding −0.8 were filtered out. Single nucleotide polymorphisms with quality by depth <2.0, FisherStrand >60.0, RMSMappingQuality <40.0, mapping quality rank sum <−12.5, and ReadPosRankSum <−8.0 were filtered out. Variants were annotated using ANNOVAR (19).

### Variant filtering, classification, and analysis

Variants with a minor allele frequency (MAF) >1 in 10,000 in the Exome Aggregation Consortium (ExAC) (20), synonymous variants not located at splice sites and nontruncating variants in *TTN* were excluded (21). Variants that remained were classified manually as pathogenic, likely pathogenic, or as variant of unknown significance (VUS) using the recent stringent ACMG consensus statement guidelines (12). Variants were checked for prior reports in the literature and in-house databases of primary electrical diseases and hereditary cardiomyopathies at the Academic Medical Centre. The reported phenotype (including diagnosis, ECGs, and cardiac symptoms), the presence of cosegregation data, and the mode of inheritance were critically assessed. Variants were then adjudicated by 2 further independent observers and agreed by consensus.

Statistical enrichment of rare variants was analyzed for in SADS cases versus controls using the sequence kernel association test (22) as implemented in RVTESTS (rare variant tests) (23). We restricted the analysis to rare variants (MAF <1 in 10,000 in ExAC) predicted to be deleterious by a Combined Annotation-Dependent Depletion (24) score >25 to decrease genetic background noise and enrich for variants that could potentially be disease causing. SADS cases and controls of non-European descent were excluded from the analysis. As a negative control, we performed the gene-based analysis using only rare synonymous variants not predicted to change the protein. Bonferroni correction for multiple testing was used to define statistical significance thresholds. We tested 77 genes and 1 phenotype (SADS): single-gene tests were considered significant if p values were < α = 0.05/77 (p < 6.5 × 10^-4^).

### Genotype-phenotype correlations

We used single and multiple logistic regression models to identify independent predictors of a positive genetic test defined as a pathogenic or likely pathogenic variant per ACMG guidelines. Variables included in the model were: sex; age category (≤35 years and >35 years); circumstances of death (adrenergic: exercise and extreme emotion; non-adrenergic: sleep, rest, and light activity); prior symptoms (transient loss of consciousness: seizures and syncope; other: palpitations and chest pain); and a family history of SCD <50 years of age. Comparison was performed using the Student *t* test for normally distributed continuous variables, the Mann-Whitney U test for non-normally distributed continuous variables, and the chi-square or Fisher’s exact test, as suitable, for categorical variables. All statistical analyses were performed using R (version 3.2.1.), and a p < 0.05 was used to indicate statistical significance.

### Family studies

Where families of SADS cases had undergone clinical evaluation and/or genetic testing (n = 82) data on clinical testing performed, clinical diagnoses and genetic testing results were gathered where available. Diagnoses were based on the current consensus guidelines 4, 25. We then assessed the independent value of molecular autopsy and familial evaluation for a familial diagnosis.

## Results

**Figure 1:**
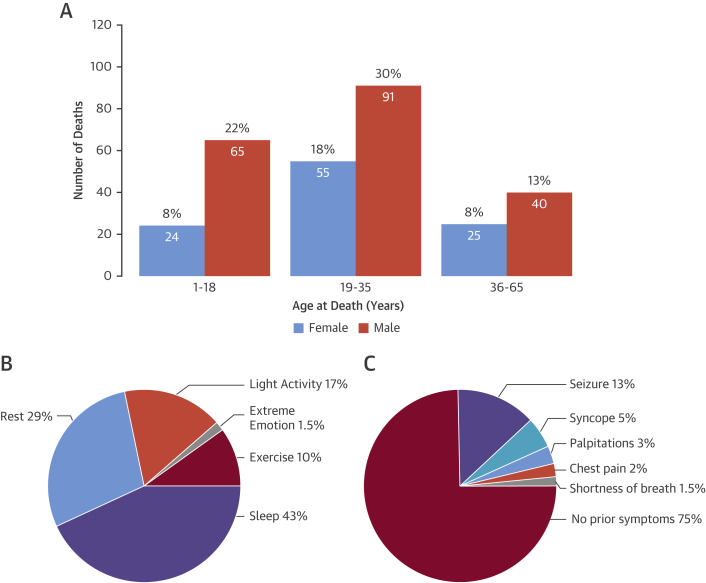
SADS: Demographic and Clinical Characterization Sudden arrhythmic death syndrome (SADS) **(A)** more often affected males and individuals 35 years and younger; **(B)** 72% of SADS deaths occurred during sleep or rest. **(C)** Whereas three-quarters exhibited no symptoms before death, 13% had a history of seizures.

Demographic and clinical characteristics of all subjects are presented in Table 1. In total, 302 sudden unexplained death patients were included (197 males and 105 females; median age: 24 years [interquartile range: 17 to 33 years; range: 1 to 64 years]) (Figure 1A). Most were of European descent (88%) and 235 died before or at the age of 35 years (78%). The most prevalent circumstances of death were during sleep (43%) or rest (29%) with death occurring during exercise or extreme emotion in 10% and 1.5%, respectively (Figure 1B). A family history of sudden death before the age of 50 years was present in 7.1% of subjects. In 18%, a personal history of syncope or seizure was reported (Figure 1C). Importantly, 24 subjects had consulted a cardiologist or general practitioner regarding these symptoms. Twenty-one subjects (7%) were diagnosed either with epilepsy or had a previous history of epilepsy; 7 patients had type 1 diabetes mellitus and were being treated with insulin from early childhood or adolescence. Among the 105 female cases, 7 died suddenly during the post-partum period.

**Figure 2:**
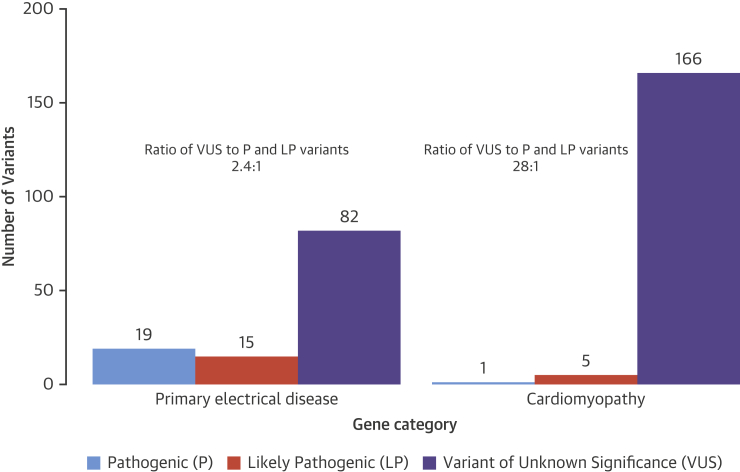
ACMG Classification of Variants A significantly higher prevalence of variants of unknown significance (VUS) was found in cardiomyopathy genes compared to primary electrical disease genes, and there was a highly unfavorable ratio of VUS to pathogenic and likely pathogenic variants in the cardiomyopathy genes (28:1) per the American College of Medical Genetics (ACMG) classification of 288 variants in 170 cases. P = pathogenic; LP = likely pathogenic.

Genetic testing was performed (Central Illustration) and there remained a total of 288 variants (MAF <1 in 10,000) in 55 different genes after variant filtering in the 302 SADS cases (Figure 2). In 132 patients (44%), no rare variant was identified. Gene level callability indicated only minor deficits or differences in coverage between cohorts (Online Table 2). Manual curation against ACMG guidelines yielded 20 pathogenic and 20 likely pathogenic variants with an overall yield of 13% (40 of 302 patients) (Table 2). Age distribution was as follows: 1 to 18 years (n = 18; 20%); 19 to 35 years (n = 13; 9%); 36 to 65 years (n = 8; 12%); and 1 age unknown. No subject carried more than 1 pathogenic or likely pathogenic variant. Nineteen pathogenic and 15 likely pathogenic variants resided in the primary electrical disorder genes, mainly *RYR2*, *KCNQ1*, *KCNH2*, and *SCN5A*. In the cardiomyopathy genes, we found 1 pathogenic variant in *PLN* and 5 likely pathogenic variants in *TTN* (n = 3), *PKP2* (n = 1), and *MYH7* (n = 1). We identified a pathogenic variant in 4 subjects >35 years of age; *KCNQ1*, *KCNH2*, *SCN5A*, and *RYR2*. Additionally, a likely pathogenic variant was identified in *KCNQ1*, *KCNH2*, *TTN*, and *MYH7* in this age category. Furthermore, we found a pathogenic variant in *RYR2* and *KCNH2* in 2 of 7 post-partum cases. Figure 2 illustrates the significantly higher prevalence of VUS in cardiomyopathy genes (97%) compared to primary electrical disease genes (71%) and the highly unfavorable ratio of VUS to pathogenic and likely pathogenic variants in the cardiomyopathy genes (28:1).

All 21 cases with epilepsy had a long-term history of syncope and seizures before death. Genetic testing identified a likely cause of death in 5 (24%): 2 pathogenic and 2 likely pathogenic variants in *RYR2* and 1 pathogenic variant in *KCNH2*.

Gene-based rare variant association analysis was undertaken in 270 SADS cases and 508 healthy controls (226 males and 282 females; mean age 42.00 ± 13.42 years; range 19 to 77 years) of European descent. Fifty-seven genes that carried 1 or more variants with MAF <1 in 10,000 and Combined Annotation-Dependent Depletion score >25 were analyzed (Online Table 4). Only *RYR2* displayed an enrichment of rare variants in cases compared to controls at our pre-specified statistical significance threshold (p = 6.5 × 10^–4^). To ensure that this result was not a consequence of systematic sequencing differences between cases and controls, we performed the same analysis using only rare synonymous variants, which did not uncover any differences (p = 0.89).

Predictors of a positive genetic test are listed in Table 3. In the single regression model, both adrenergic circumstances of death (exercise and extreme emotion; odds ratio [OR]: 4.33; 95% confidence interval [CI]: 1.77 to 10.21; p = 0.0009), and a positive family history of SCD <50 years (OR: 4.45; 95% CI: 1.55 to 12.00; p = 0.004), were associated with a positive genetic test. In the multiple logistic regression model, adrenergic circumstances of death (exercise and extreme emotion; OR: 4.38; 95% CI: 1.63 to 11.28; p = 0.002) was independently associated with a positive genetic test. Neither sex nor age category at death (≤35 years or >35 years) were associated with a positive genetic test. Using age as a quantitative trait in the analyses did not change the results.

Genotype-phenotype correlations in SADS cases with *RYR2* variants are shown in Table 1. Since a substantial number of SADS cases (n = 17) had pathogenic/likely pathogenic variants in *RYR2*, we compared their characteristics with the remaining patients. *RYR2* cases were significantly younger (median age 13 years versus 25 years; p = 0.0004) in comparison to cases with negative *RYR2* testing (n = 285) and death occurred more often during exercise or extreme emotion (9 of 17 vs. 21 of 245; p < 0.0001). Of the 4 SADS subjects who died suddenly during extreme emotion, all carried a pathogenic or likely pathogenic variant in *RYR2*. There was no statistically significant difference in family history of sudden death between *RYR2* cases as compared to the rest of the SADS cohort (3 of 15 vs. 16 of 254; p = 0.0791).

Cardiac evaluation was performed in 282 relatives from 82 SADS families (Central Illustration). Family members underwent 12-lead ECG (n = 282), signal-averaged ECG (n = 85), exercise testing (n = 192), 24-h Holter monitoring (n = 178), transthoracic 2-dimensional echocardiograms (n = 206), ajmaline/flecainide testing (n = 101), and cardiac magnetic resonance imaging (n = 41) in expert centers. Thirty-five relatives (12%) from 21 (26%) families were clinically diagnosed with a primary electrical disease: BrS in 20 relatives from 14 families (17%); CPVT in 12 from 4 families (5%); and LQTS in 5 relatives from 3 families (4%). Relatives received treatment per current guidelines (4).

We then considered the added diagnostic value of combining clinical evaluation of family members and post-mortem genetic testing in the SADS case (Central Illustration). Molecular autopsy had identified a pathogenic or likely pathogenic variant in 18 of 82 families (22%). Relevant pedigrees are shown in the Online Appendix. Eight variants (10%) were considered diagnostic for CPVT, 5 (6%) for LQTS, 2 for BrS (2%), and 3 (3.5%) for cardiomyopathy. An overlap was observed in 7 of 82 families (8.5%) where molecular autopsy and clinical evaluation of family members resulted in the same diagnosis: CPVT in 4, LQTS in 2, and BrS in 1 (see Online Figure 1, Family 6 for segregation of *SCN5A* variant). Therefore, an additive independent yield of molecular autopsy was seen in 11 of 82 families (13%). In 14 families (17%), clinical screening of relatives identified a clinical diagnosis that was not confirmed by post-mortem genetic screening in the index SADS case; 13 of these were diagnosed with BrS whereas 1 family was diagnosed with LQTS. Overall, the combined diagnostic yield of clinical evaluation of family members and molecular autopsy in the index SADS case was 32 of 82 families (39%; 95% CI: 28% to 49%). In 2 SADS cases, combined clinical and genetic screening of surviving relatives led to an upgrade of a *RYR2* variant ACMG classification from likely pathogenic to pathogenic because of de novo inheritance in 1 family (Online Figure 1, Family 3) and clear cosegregation with exertional arrhythmia in the other (Online Figure 1, Family 5). In a 4-year-old SADS case, a homozygous nonsense variant was identified in *KCNQ1*. Re-examination of the medical history confirmed that the patients’ parents were consanguineous and that the deceased had a history of congenital sensorineural deafness, confirming a clinical diagnosis of Jervell and Lange-Nielsen syndrome (Online Figure 1, Family 1).

## Discussion

We report, to our knowledge, the largest study of molecular autopsy and its clinical utility in a set of 302 stringently phenotyped SADS cases screened for a large panel of 77 genes using next-generation sequencing. Applying highly stringent criteria as indicated by the ACMG guidelines, we identified a pathogenic or likely pathogenic variant, which we consider actionable, in 13% of cases. The main causes of death established through genetic testing were CPVT and LQTS. This was further supported by the case-control rare variant association analysis we conducted, which demonstrated enrichment of rare predicted deleterious variants in the *RYR2* gene responsible for CPVT. We combined molecular autopsy with clinical evaluation in surviving family members and demonstrated the added value of such an approach.

### Comparison with previous next-generation sequencing molecular autopsy studies

Our study identified a modest, though likely more realistic, yield of genetic testing (13%) in SADS cases, compared to previous studies reporting a yield of 27% to 32% 26, 27, 28. These earlier studies had relied strongly on the application of MAF thresholds and estimation of pathogenicity by computational approaches in determining yield. Often the MAF threshold used had been too lenient when the Mendelian nature of the underlying disorders is considered and that variants that contribute to such disorders are expected to be very rare in the general population. In our study, besides applying a stringent MAF threshold of 1 in 10,000, which we have previously validated as appropriate for these conditions (11), we strove to achieve robust and rigorous variant classification by evaluating each variant against the ACMG variant classification guidelines. Admissible evidence included family segregation data; population data (such as novelty); observations in multiple, unrelated, clinically affected individuals; functional evidence; and concordance between mutation type and genetic mechanism of the disease. Despite this there is still an element of subjectivity that may result in variability of final assignment by different laboratories. Specifically, Bagnall et al. (1) reported a 27% yield of “clinically relevant” cardiac genetic variants. Importantly, clinical relevance was partially based on in silico prediction tools, which may have led to an overestimation of the yield. Indeed, our lower yield of genetic testing is similar to that found in a previous smaller study that reported an overall yield of 7%; this study tested a comparable number of genes and also applied ACMG guidelines (10).

Our study found a likely pathogenic or pathogenic *RYR2* variant in 17 patients (6%) in whom death occurred at a significantly younger age and more often during exercise or extreme emotion. This was consistent with our previous characterization of SADS cases that asserted that CPVT was an important cause of death in the young (29). The predominant role of *RYR2* was further illustrated by enrichment of rare, highly deleterious *RYR2* variants in SADS cases compared to controls.

A subanalysis of 82 families showed that combining molecular autopsy with comprehensive clinical screening of surviving relatives increased the diagnostic yield to 39% indicating the potential complementary value of both approaches when undertaken systematically hand-in-hand 4, 9. Of 14 families with a negative molecular autopsy, clinical assessment of relatives uncovered a diagnosis of BrS in 13 cases. These diagnoses were in keeping with guidelines for investigation of SADS families (4) and the recent consensus document on diagnosis in BrS (25). The proper role of provocation testing in making these diagnoses does, however, require further clarification (25). Genetic variation in *SCN5A* is found in only ∼20% of BrS cases and, although variants in other genes have been implicated, these collectively account for <1% of cases. Recently, complex inheritance has been proposed for the disorder 5, 30. Thus, the diagnosis rests on clinical evaluation.

Some of the previous post-mortem genetic studies have only included SADS patients who were <35 years of age 1, 2, 8. However, our previous work has shown no significant difference between the characteristics of younger and older adults with SADS (29). We included SADS cases from 35 to 65 years of age and identified a pathogenic or likely pathogenic variant in 7. In 1 case, the family history included 2 additional SADS cases (death at ages 14 and 41 years old, respectively) with post-mortem DNA available (Online Figure 1, Family 10). The *RYR2* variant identified in the proband segregated perfectly in this family and identified multiple clinically affected and living patients requiring treatment per current CPVT guidelines. In line with these data, age was not an independent predictor of a pathogenic or likely pathogenic variant in the logistic regression model (Table 3). Therefore, our data indicates a role for molecular autopsy beyond 35 years of age 4, 9, although these results could have been biased. Older SCDs are less likely to be referred for autopsy, and those who are referred may tend to have a family history of SCD and, thus, be enriched for genetic risk. Furthermore, the limited number of patients >50 years old included in this study probably resulted in a lack of power to detect a difference in this age category.

### Warning symptoms before death

One in 5 subjects had experienced a syncope or seizure before death and 24 were seen by a physician for these symptoms. In 2 children in whom we identified a pathogenic variant in *RYR2*, an antemortem resting ECG had shown no abnormalities. Unfortunately, no exercise testing had been performed. In one-quarter of cases diagnosed with epilepsy, molecular autopsy identified a likely cause of death, consistent with previous studies indicating that CPVT or LQTS patients can be misdiagnosed with epilepsy and drug-resistant epilepsy (31). These data emphasized the importance of thorough clinical and cardiac examination, including exercise testing of patients presenting with syncope or seizures, especially during exercise (32).

### Cardiomyopathy-associated variants in SADS

The significance of the cardiomyopathy-associated genes in SADS with normal cardiac autopsy is largely unexplored. Previous studies that expanded the number of genes in molecular autopsy panels uncovered a large number of cardiomyopathic rare variants that appeared to enhance the overall yield but at a cost of increased VUS (8). Indeed, the ratio of rare VUS to pathogenic or likely pathogenic cardiomyopathic variants was extremely unfavorable in our cohort (Figure 2) and illustrated the need for careful and stringent adjudication. Nonetheless, through careful use of the ACMG criteria, we still identified 6 pathogenic or likely pathogenic variants in cardiomyopathy genes. This confirmed findings of heritable cardiomyopathy in SADS families following clinical evaluation (6) and may be due to subtle and perhaps localized structural disease not noticeable at autopsy or, alternatively, to effects of genetic variation in these genes on cardiac electrical function before the onset of cardiomyopathic changes. For example, electrophysiological changes, including a decreased sodium current, were observed in the absence of overt cardiomyopathy in mouse models of desmosomal arrhythmogenic cardiomyopathy 33, 34.

### Study limitations

In most SADS cases included in this study (220 of 302; 73%) we were not able to perform comprehensive clinical evaluation of relatives. Therefore, the combined value of genetic testing and clinical evaluation of relatives could only be assessed in 82 families. Antemortem data were missing in 40 cases, which may have affected genotype and phenotype correlations minimally. While included cases were either consecutive referrals or from population studies, we recognize that there may be an element of referral bias.

A small number of genotype-positive LQTS cases from New Zealand were not included in the study 13, 14. Of these, only 3 would have been adjudicated as pathogenic or likely pathogenic by ACMG criteria and would not have affected the yield significantly. In addition, some exons were moderately covered (e.g., exon 1 of *KCNQ1* and *KCNH2* in cohort 1) (Online Table 3), but this was unlikely to have resulted in significant underestimation of the yield. Interpretation of variants in the small minority of non-Caucasian cases may have been hampered by the absence of large, ethnically specific population databases to which we could refer. Furthermore, we did not sequence genes currently associated with noncardiac disorders that predispose to sudden death.

## Conclusions

Next-generation sequencing based molecular autopsy identified a 13% yield of clinically actionable rare variants in SADS cases. This required the application of a stringent allele frequency threshold and ACMG guidelines to achieve robust, clinically useful results. Furthermore, its systematic use, combined with clinical and genetic evaluation of families, enhanced diagnostic utility. Our data also highlighted the important role of *RYR2* in SADS. Molecular autopsy should, however, not be limited to only young cases, as the yield is independent of age at death.

Perspectives **COMPETENCY IN MEDICAL KNOWLEDGE:** Post-mortem genetic testing can establish a molecular diagnosis in a substantial minority of cases of SADS. When combined with clinical evaluation of relatives, such testing increases the diagnostic yield to approximately 40%, but most cases of SADS cannot be explained by clinically detectable genetic disorders in family members.

**TRANSLATIONAL OUTLOOK:** Future research into the causes of SADS should include not only family and functional studies but also nongenetic and noncardiac etiologies such as neurological disease and environmental triggers of arrhythmias.

## Figures and Tables

**Central Illustration:**
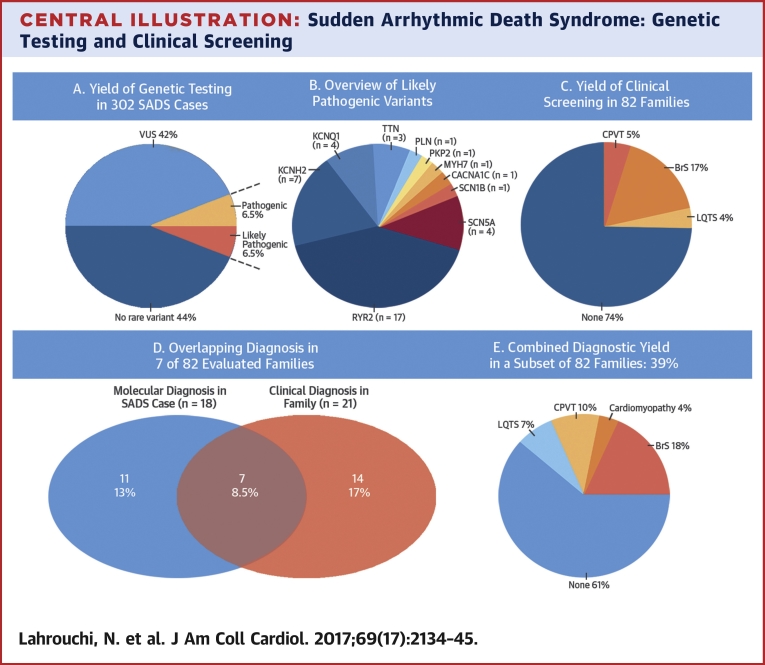
Sudden Arrhythmic Death Syndrome: Genetic Testing and Clinical Screening **(A to E)** Genetic testing via next-generation sequencing in 302 sudden arrhythmic death syndrome (SADS) cases identified pathogenic and likely pathogenic variants in 13% of cases. Clinical screening of relatives in 82 families found 35 relatives from 21 families with a clinical diagnosis of a primary electrical disease: Brugada syndrome (BrS); catecholaminergic polymorphic ventricular tachycardia (CPVT); and long-QT syndrome (LQTS); there was overlap between molecular and clinical diagnosis in 7 families. Combining molecular autopsy with clinical evaluation increased diagnostic yield in surviving families to 39%. VUS = variant of unknown significance.

**Table 1:** Demographic and Genotype-Phenotype Correlations of the SADS Cohort

	SADS (n = 302)	SADS and Negative Genetic Testing (n = 262)∗	SADS and Positive Genetic Testing (n = 40)∗	SADS Without Positive Genetic Testing for a *RYR2* Variant (n = 285)∗	SADS and Positive Genetic Testing for a *RYR2* Variant (n = 17)∗	p Value†
Age at death, yrs	24 (17–33)	25 (18–33)	20 (11–32)	25 (18–34)	13 (8–18)	0.0004
Male	197/302 (65)	172/262 (66)	25/40 (62)	183/285 (64)	14/17 (82)	0.1918
Circumstances of death‡						
Exercise and extreme emotion	30/262 (11)	20/228 (9)	10/34 (29)	21/245 (9)	9/17 (53)	<0.0001
Sleep and rest	188/262 (72)	170/228 (75)	18/34 (53)	183/245 (75)	5/17 (29)	0.0002
Light activity	44/262 (17)	38/228 (17)	6/34 (18)	41/245 (17)	3/17 (18)	1.0000
Symptoms before death‡						
Syncope and/or seizures	50/268 (18)	40/235 (17)	10/33 (30)	43/253 (17)	7/15 (47)	0.0101
Other§	18/268 (7)	16/235 (7)	2/33 (6)	17/253 (7)	1/15 (7)	1.0000
None	200/268 (75)	179/235 (76)	21/33 (64)	193/253 (76)	7/15 (47)	0.0269
Family history of SCD <50 yrs of age‡	19/269 (7)	12/233 (5)	7/36 (19)	16/254 (6)	3/15 (20)	0.0791

Values are median (interquartile range) or n/N (%).

ACMG = American College of Medical Genetics; SADS = sudden arrhythmic death syndrome; SCD = sudden cardiac death.

∗ Positive outcome was defined as a pathogenic or likely pathogenic variant per the ACMG guidelines (12).

† p values refer to the following comparison: positive genetic testing *RYR2* (n = 17) versus no positive *RYR2* test (n = 285).

‡ Note missing data for these parameters and that percentages are based on nonmissing data.

§ Includes palpitations, shortness of breath, and chest pain.

**Table 2:** Overview of Pathogenic and Likely Pathogenic Variants in SADS Cohort

Case	Age at Death (yrs)	Sex	Circumstances of Death	Symptoms Before Death	Gene	Chr	Start	Ref	Alt	cDNA change	Amino Acid Change∗	ACMG Classification†	ACMG Criteria†
P1	31	M	NA	NA	*KCNH2*	7	150671971	G	C	c.C135G	p.N45K	LP	PM1,PM2,PM5,PP3
P2	22	M	Rest	None	*KCNH2*	7	150655191	A	G	c.T872C	p.M291T	LP	PS4,PM2
P3	19	F	Sleep	None	*KCNH2*	7	150649550	G	A	c.C1520T	p.P507L	P	PM1,PM2,PM5,PP3
P4	44	F	Rest	None	*KCNH2*	7	150649880	C	T	c.G1190A	p.R397H	LP	PS3,PM5,PP3
P5	22	F	Sleep	None	*KCNH2*	7	150647481	G	A	c.C2173T	p.Q725X	P	PVS1,PM2,PP3
P6	22	F	NA	NA	*KCNH2*	7	150644460	GC	G	c.3107_3108C	p.G1036AfsX21	P	PVS1,PM2,PP1,PP3
P7	39	F	Sleep	Seizures	*KCNH2*	7	150648593	C	T	c.G1888A	p.V630I	P	PS4,PM1,PM5,PP3,PP5
P8	10	M	Light activity	Seizures	*KCNQ1*	11	2466615	C	G	c.C287G	p.T96R	LP	PS3,PM2
P9	57	M	Sleep	None	*KCNQ1*	11	2610045	C	T	c.C1354T	p.R452W	LP	PS4,PM5,PP3
P10	42	F	Sleep	None	*KCNQ1*	11	2797262	C	T	c.C1663T	p.R555C	P	PS3,PS4,PP3
P11	4	F	Light activity	NA	*KCNQ1*	11	2604712	G	A	c.G969A (homozygous)	p.W323X (homozygous)	P	PVS1,PM2,PM3,PP3
P12	1	M	Sleep	None	*CACNA1C*	12	2789725	G	GC	c.5608_5608delinsGC	p.Q1872PfsX24	LP	PVS1,PM2
P13	20	M	Sleep	None	*RYR2*	1	237586538	G	A	c.G995A	p.R332Q	P	PS4,PM1,PM2,PM5,PP3
P14	18	M	Extreme emotion	Syncope	*RYR2*	1	237608789	G	A	c.G1259A	p.R420Q	P	PS4,PS3,PM1,PM2,PM5,PP3
P15	17	M	Exercise	None	*RYR2*	1	237617856	A	C	c.A1458C	p.Q486H	LP	PM2,PP3,PP4‡
P16	15	M	Exercise	Seizures	*RYR2*	1	237754262	C	T	c.C4130T	p.A1377V	LP	PM2,PP3,PP4‡
P17	12	M	Exercise	None	*RYR2*	1	237777676	G	A	c.G5248A	p.G1750R	LP	PM2,PP3,PP4‡
P18	8	M	Exercise	None	*RYR2*	1	237794789	A	G	c.A6503G	p.H2168R	LP	PM2,PM5,PP3,PP4
P19	12	F	Light activity	Syncope	*RYR2*	1	237801693	T	G	c.T6829G	p.C2277G	LP	PM1,PM2,PM5,PP3
P20	6	M	Exercise	Syncope	*RYR2*	1	237804283	G	A	c.G7202A	p.R2401H	P	PS4,PM1,PM2,PM5,PP3
P21	11	M	Extreme emotion	None	*RYR2*	1	237870332	G	A	c.G9664A	p.A3222T	LP	PM2,PM6,PP2,PP3
P22	22	F	Extreme emotion	NA	*RYR2*	1	237538090	C	T	c.C458T	p.T153I	P	PS4,PM1,PP3‡
P23	24	M	Rest	None	*RYR2*	1	237538090	C	T	c.C458T	p.T153I	P	PS4,PM1,PP3‡
P24	18	M	Rest	None	*RYR2*	1	237886554	C	G	c.C10681G	p.L3561V	LP	PM1,PM2,PP3‡
P25	8	M	Extreme emotion	Seizures	*RYR2*	1	237942026	G	A	c.G11836A	p.G3946S	P	PS4,PM1,PM2,PM5,PP3
P26	3	F	Rest	NA	*RYR2*	1	237947016	A	G	c.A12004G	p.M4002V	P	PS2,PM1,PM2,PM5,PP3
P27	13	M	Sleep	Seizures	*RYR2*	1	237947164	C	T	c.C12152T	p.A4051V	LP	PM1,PM2,PP3
P28	6	M	Light activity	Syncope	*RYR2*	1	237957170	C	T	c.C13786T	p.P4596S	P	PM1,PM2,PP1,PP3,PP4
P29	41	M	Light activity	Chest pain	*RYR2*	1	237957207	G	A	c.G13823A	p.R4608Q	P	PS4,PM1,PM2,PM5,PP1,PP3
P30	2	M	Sleep	None	*SCN5A*	3	38627337	G	A	c.C2632T	p.R878C	P	PS4,PS3,PM2,PM5,PP3
P31	1	M	Sleep	None	*SCN5A*	3	38620964	C	T	c.G3248A	p.G1083D	LP	PS4,PM2
P32	39	M	NA	None	*SCN5A*	3	38671833	G	A	c.C361T	p.R121W	P	PS3,PS4,PM2,PP1,PP3
P33	24	F	Light activity	None	*SCN5A*	3	38655264	G	A	c.C673T	p.R225W	P	PS3,PS4
P34	21	F	NA	NA	*SCN1B*	19	35524731	G	A	c.G536A	p.W179X	P	PVS1,PS1,PS4,PS3
P35	55	F	Rest	None	*TTN*	2	179391915	C	A	c.G80605T	p.G26869X	LP	PVS1,PM2
P36	33	F	NA	Seizures	*TTN*	2	179640969	C	T	c.G5484A	p.W1828X	LP	PVS1, PS4‡
P37	NA	F	NA	NA	*TTN*	2	179640969	C	T	c.G5484A	p.W1828X	LP	PVS1, PS4‡
P38	32	M	Rest	None	*PLN*	6	118880200	T	G	c.T116G	p.L39X	P	PVS1,PS4,PP5
P39	22	M	Exercise	None	*PKP2*	12	32975414	CT	C	c.1825_1826G	p.R609GfsX3	LP	PVS1,PM2
P40	54	M	Sleep	Palpitations	*MYH7*	14	23886806	C	T	c.G4259A	p.R1420Q	LP	PS4,PM2,PM5,PP3

Alt = alternate allele; cDNA = coding DNA; Chr = chromosome; LP = likely pathogenic; NA = not available; P = pathogenic; Ref = reference allele; other abbreviations as in Table 1.

∗ The following transcripts were used: *CACNA1C*:NM_001167625; *KCNH2*:NM_000238; *KCNQ1*:NM_000218; *PKP2*:NM_001005242; *PLN*:NM_002667; *RYR2*:NM_001035; *SCN1B*:NM_199037; *SCN5A*:NM_000335; *TTN*:NM_003319.

† See ACMG guidelines (12) for further information on classification criteria.

‡ For these variants, 1 criterion was moved to a higher weight using consensus and professional judgment of 3 observers, in line with the ACMG guidelines.

**Table 3:** Association of Predictors and Positive Post-Mortem Genetic Testing

Predictor	Positive Genetic Testing		Multiple Regression Model		Single Regression Model	
	Predictor Present (%)	Predictor Absent (%)	OR (95% CI)	p Value	OR (95% CI)	p Value
Death ≤35 yrs of age	13	12	NA	NA	1.08 (0.49–2.64)	0.851
Male	1	14	NA	NA	0.87 (0.44–1.77)	0.697
Death during adrenergic circumstances (exercise and extreme emotion)	33	10	4.38 (1.63–11.28)	0.002	4.33 (1.77–10.21)	0.0009
TLOC (seizures and syncope) before death	20	11	2.42 (0.94–5.90)	0.057	2.12 (0.90–4.70)	0.071
Family history of SCD <50 yrs of age	37	12	3.16 (0.84–10.52)	0.069	4.45 (1.55–12.00)	0.004

CI = confidence interval; OR = odds ratio; TLOC = transient loss of consciousness; other abbreviations as in Tables 1 and 2.
