# Supplementary material for: Utility of Post-Mortem Genetic Testing in Cases of Sudden Arrhythmic Death Syndrome
Source: J Am Coll Cardiol. 2017 May 2;69(17):2134–45. doi: 10.1016/j.jacc.2017.02.046 (PMC5405216; doi:10.1016/j.jacc.2017.02.046)
Supplement: Online Data [file mmc1.doc]

Online Appendix

**Utility of Post-mortem Genetic Testing in Cases of Sudden Arrhythmic Death Syndrome**

Najim Lahrouchi, MD, Hariharan Raju, MBChB, PhD, Elisabeth M. Lodder, PhD, Efstathios Papatheodorou, MD, James S. Ware, PhD, MRCP, Michael Papadakis, MBBS, MD, Rafik Tadros, MD, PhD, Della Cole, BSc, Jonathan R. Skinner, MBChB, MD, Jackie Crawford, NZCS, Donald R. Love, PhD, Chee J. Pua, PhD, Bee Y. Soh, PhD, Jaydutt D. Bhalshankar, PhD, Risha Govind, MSc, Jacob Tfelt-Hansen, MD, DMSc, Bo G. Winkel, MD, PhD, Christian van der Werf, MD, PhD, Yanushi D. Wijeyeratne, BMBS, Greg Mellor, MBChB, MD, Jan Till, MD, Marta C. Cohen, MD, FRCPath, DMJ (Pathol), Maria Tome-Esteban, MD, PhD, Sanjay Sharma, MBChB, MD, Arthur A.M. Wilde, MD, PhD, Stuart A. Cook, MD, PhD, Connie R. Bezzina, PhD, Mary N. Sheppard, MB, BCh, BAO, MD,

Elijah R. Behr, MBBS, MD

**TABLE OF CONTENT**

Page

- Legend Online Figure 1 3
- Online Figure 1 7
- Online Table 1 8
- Online Table 2 15
- Online Table 3 17
- Online Table 4 19
- Online References 22

**LEGEND** **ONLINE FIGURE 1**.

Selection of pedigrees of families investigated for sudden arrhythmic death syndrome (SADS). Legend:

**Family 1.** Post-mortem genetic testing in the 4-year-old deceased proband (arrow) identified a homozygous pathogenic nonsense variant in *KCNQ1*. Review of the medical history of the deceased revealed that the patient was deaf since birth establishing the diagnosis of Jervell and Lange-Nielsen syndrome. Both consanguineous parents and two siblings carried the *KCNQ1* p.W323X variant in heterozygous state. Clinical testing of heterozygous carriers did not reveal any abnormalities on ECG or during exercise testing.

**Family 2.** Post-mortem genetic testing in the 21-year old deceased proband (arrow) identified a frameshift variant (p.G696AfsX21) in *KCNH2*. Clinical assessment and genetic testing in the family identified a prolonged QTc interval (490ms) in the mother and grandmother who turned out to carry the same variant as the deceased. The mother of the proband had suffered from syncope during pregnancy. The younger sibling of the deceased was clinically unaffected (e.g. normal QTc-interval on ECG and normal exercise testing) and turned out not to carry the *KCNH2* variant. Treatment with beta-blocker has been initiated in the mother.

**Family 3.** Post-mortem genetic testing in the 3-year old deceased proband (arrow) identified a *de novo* variant in *RYR2* (p.M4002V). Both parents are unaffected and do not carry the *RYR2* variant.

**Family 4.** Post-mortem genetic testing in the 21-year old deceased proband (arrow) identified a pathogenic variant in *SCN1B* (p.W179X). No evidence of a cardiac disorder was demonstrated in either family member following comprehensive cardiac testing involving an ECG, echocardiogram, exercise test, 24-hour holter testing and an Ajmaline provocation test. Genetic testing of relatives is currently being performed.

**Family 5.** Post-mortem genetic testing in the 6-year old deceased proband (arrow) identified a likely pathogenic variant in *RYR2* (p.P4596S). The deceased had experienced two episodes of syncope during exertion. ECG and EEG testing was undertaken and a diagnosis of fainting was made. Both parents and his sister underwent thorough clinical screening. The father reported occasional episodes of palpitations accompanied by dizziness but denied having syncope or seizure. Exercise testing in the father revealed ventricular ectopy in a bigeminal pattern which became increasingly frequent with bi-directional couplets, atrial fibrillation, eventually returning to normal sinus rhythm in recovery. Clinical examination of the mother was normal. The deceased’s 10-year-old sister was asymptomatic. Her ambulatory, exercise and epinephrine tests did not reveal any arrhythmias. The father was treated with a betablocker and flecainide and found to be a carrier.

**Family 6.** Post-mortem genetic testing in the 39-year old deceased proband (arrow) identified a pathogenic variant in *SCN5A* (p.R121W). The deceased did not have any cardiac symptoms prior to death. His mother had a history of palpitations. Her resting ECG showed a type 2 Brugada pattern with minimal J-point elevation, saddleback shaped ST segment elevation and T wave inversion in the higher right precordial leads. During the ajmaline provocation test, she developed an unequivocal type 1 Brugada pattern in the higher right precordials leads. The brother of the deceased also underwent an Ajmaline provocation test which revealed a type I Brugada ECG pattern. His resting ECG showed evidence of first degree AV block and slight interventricular conduction delay. Both were carriers.

**Family 7.** Post-mortem genetic testing in the 24-year old deceased proband (arrow) identified a pathogenic variant in *RYR2* (p.T153I). The deceased had complained of palpitations prior to his death although these were not investigated. He had not had any episode of dizziness or syncope. Both the mother and brother of the deceased attended the familial cardiac screening following the death of their relative. No evidence of a cardiac disorder was demonstrated in either family member following comprehensive cardiac testing involving an ECG, echocardiogram, exercise test, 24-hour holter testing and an Ajmaline provocation test. His 2-year-old son has been tested by a paediatric cardiologist and no abnormality has been found. The deceased father has not had any cardiac investigations. Genetic screening of family members is awaited.

**Family 8.** Post-mortem genetic testing in the 6-year old deceased proband (arrow) identified a pathogenic variant in *RYR2* (p.R2401H). The deceased had an impressive history of seizures during exercise and had been suspected of having epilepsy. Both siblings and parents underwent thorough clinical screening which did not reveal any abnormalities. There was no other history of syncope or seizures in the family. The parents declined further genetic testing and therefore the inheritance pattern of this variant in the family remains unknown.

**Family 9**. Post-mortem genetic testing in the 11-year old deceased proband (arrow) identified a likely pathogenic variant in *RYR2* (p.A3222T). The deceased had been in a fight with a peer after which he collapsed and lost consciousness. CPR was started and VF had been registered but resuscitation failed. His older sister and mother underwent extensive clinical screening (ECG, exercise ECG, Holter, echocardiogram) which did not show any abnormalities. The father had committed suicide 1 year prior to the death of his son. He did not have any episodes of syncope and was in good clinical health. The mother declined further genetic testing and therefore the inheritance pattern of this variants in the family remains unknown.

**Family 10.** Post-mortem genetic testing in the 40-year old deceased proband (arrow) identified a pathogenic variant in *RYR2* (p.R4608Q). The deceased had a positive family history of sudden unexplained death in a 14-year old cousin. Unfortunately during clinical assessment of the family one additional event of sudden death occurred in a 40-year old cousin. Genetic testing in the deceased cousin using DNA extracted from post-mortem tissue identified the same *RYR2* variant as the patient included in our study. Further genetic assessment of the family discovered multiple carriers of the *RYR2* variant. Clinical and genetic screening of family members is currently being performed.

**Family 11.** Post-mortem genetic testing in the 11-year old deceased proband (arrow) identified a variant in *KCNQ1* (p.T96R). His older sister and mother, who are also carriers underwent clinical screening with a QTc of 400ms and 450ms respectively. The father had a QTc of 400ms. In vitro, electrophysiological testing was performed using whole-cell patch clamping which showed that the mutant channel protein function was unambiguously abnormal, with a severe reduction of total currents and a positive shift of voltage dependence of activation (**see reference 1**)

**ONLINE FIGURE 1**


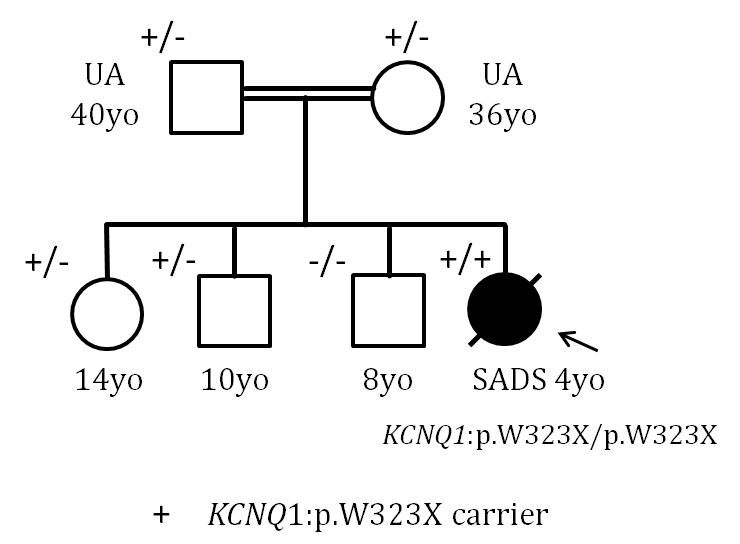

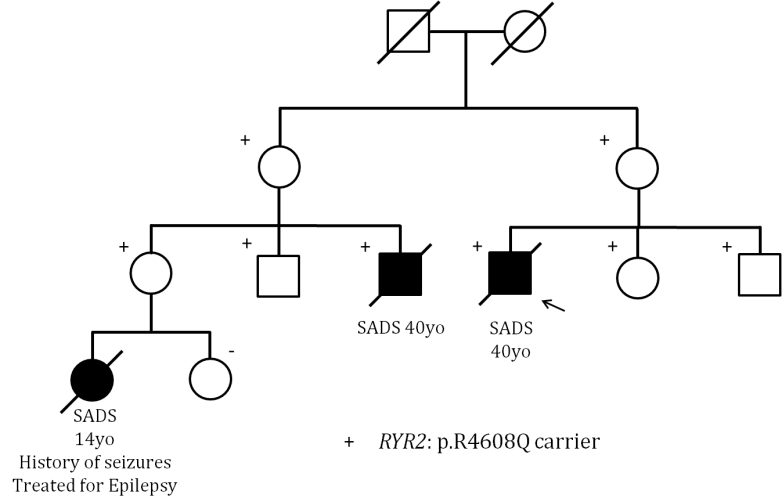

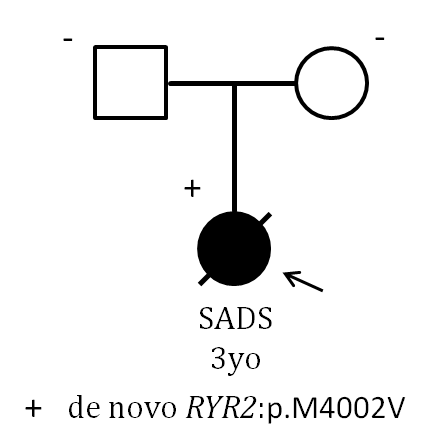

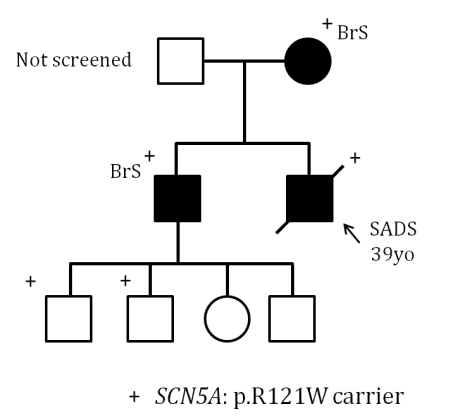

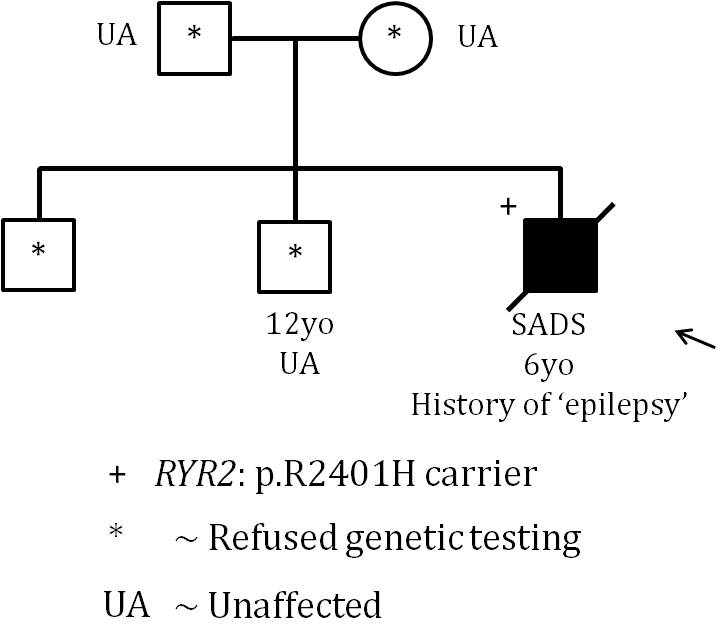

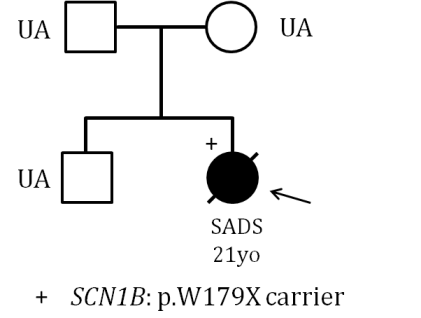

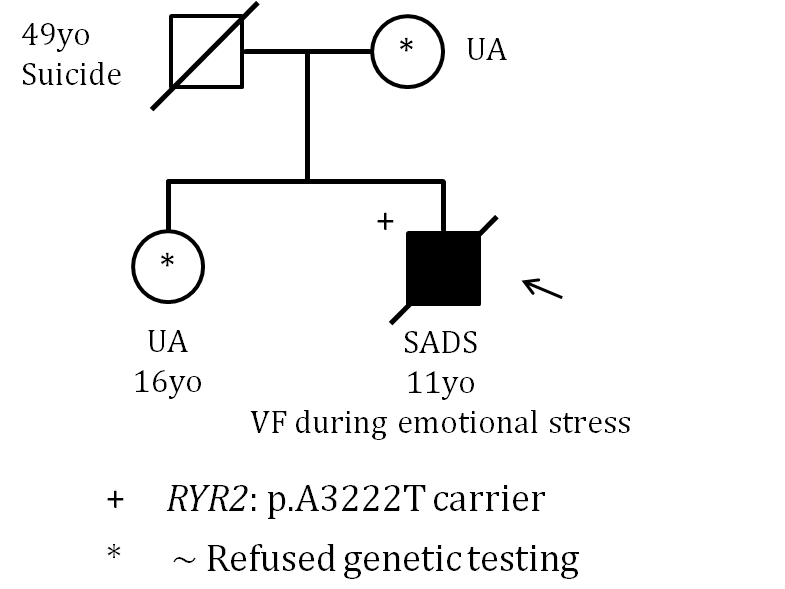


Family 1

Family 2

Family 3

Family 4

Family 5

Family 6

Family 11

Family 10

Family 7

Family 8

Family 9


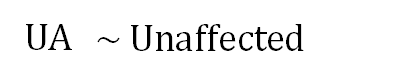

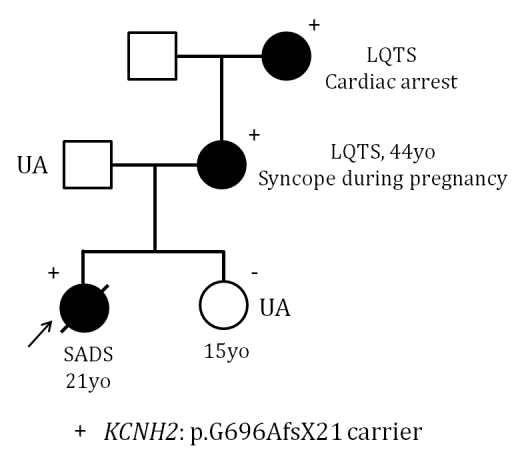

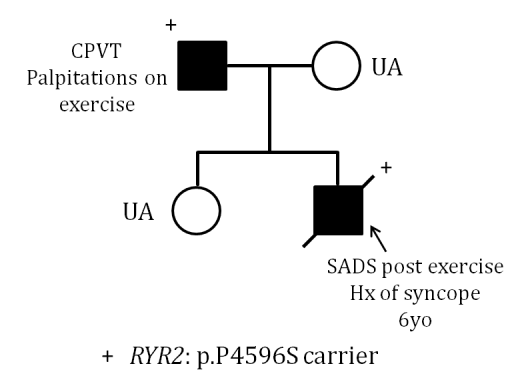

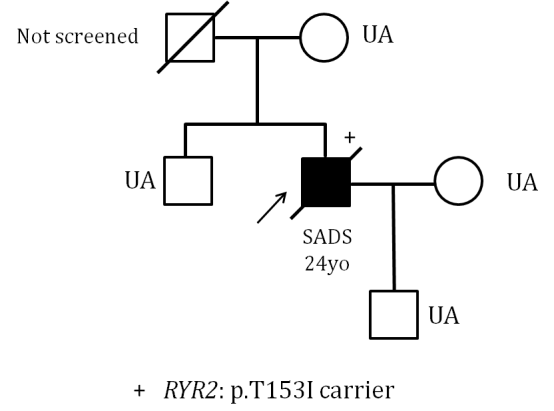

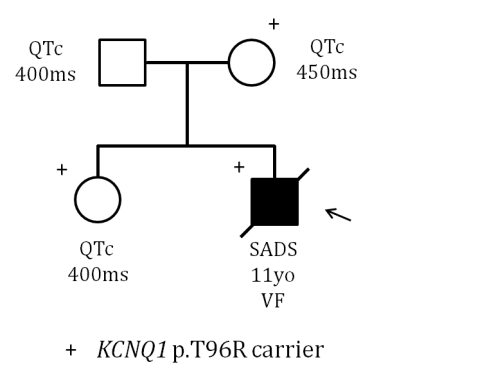


**ONLINE TABLE 1**

**List of 77 genes tested in Sudden Arrhythmic Death Syndrome (SADS) cohort. Adapted from reference 2.**

| **Gene** | **Gene name** |
| --- | --- |
|  |  |
| ***Inherited Arrhythmias*** | |
| *Long QT syndrome (LQTS) - 16 genes* | |
| *KCNQ1* | potassium voltage-gated channel, KQT-like subfamily, member 1 |
| *KCNH2* | potassium voltage-gated channel, subfamily H (eag-related), member 2 |
| *SCN5A* | sodium channel, voltage-gated, type V, alpha subunit |
| *ANK2* | ankyrin 2, neuronal |
| *KCNE1* | potassium voltage-gated channel, Isk-related family, member 1 |
| *KCNE2* | potassium voltage-gated channel, Isk-related family, member 2 |
| *KCNJ2* | potassium inwardly-rectifying channel, subfamily J, member 2 |
| *CACNA1C* | calcium channel, voltage-dependent, L type, alpha 1C subunit |
| *CAV3* | caveolin 3 |
| *SCN4B* | sodium channel, voltage-gated, type IV, beta |
| *AKAP9* | A kinase (PRKA) anchor protein (yotiao) 9 |
| *SNTA1* | syntrophin, alpha 1 (dystrophin-associated protein A1, 59kDa, acidic component) |
| *KCNJ5* | potassium inwardly-rectifying channel, subfamily J, member 5 |
| *RYR2* | ryanodine receptor 2 (cardiac) |
| *KCNE3* | potassium voltage-gated channel, Isk-related family, member 3 |
| *CALM1* | calmodulin 1 (phosphorylase kinase, delta) |
| *Short QT syndrome (SQTS) - 4 genes* | |
| *KCNH2* | potassium voltage-gated channel, subfamily H (eag-related), member 2 |
| *KCNQ1* | potassium voltage-gated channel, KQT-like subfamily, member 1 |
| *KCNJ2* | potassium inwardly-rectifying channel, subfamily J, member 2 |
| *CACNA2D1* | calcium channel, voltage-dependent, alpha 2/delta subunit 1 |
|  |  |
| *Brugada syndrome (BrS) - 13 genes* | |
| *SCN5A* | sodium channel, voltage-gated, type V, alpha subunit |
| *GPD1L* | glycerol-3-phosphate dehydrogenase 1-like |
| *CACNA1C* | calcium channel, voltage-dependent, L type, alpha 1C subunit |
| *CACNB2* | calcium channel, voltage-dependent, beta 2 subunit |
| *KCNE3* | potassium voltage-gated channel, Isk-related family, member 3 |
| *SCN3B* | sodium channel, voltage-gated, type III, beta |
| *KCNJ8* | potassium inwardly-rectifying channel, subfamily J, member 8 |
| *CACNA2D1* | calcium channel, voltage-dependent, alpha 2/delta subunit 1 |
| *RANGRF* | RAN guanine nucleotide release factor |
| *HCN4* | hyperpolarization activated cyclic nucleotide-gated potassium channel 4 |
| *KCNH2* | potassium voltage-gated channel, subfamily H (eag-related), member 2 |
| *PKP2* | plakophilin 2 |
| *ABCC9* | ATP-binding cassette, sub-family C (CFTR/MRP), member 9 |
| *SCN1B* | sodium channel, voltage-gated, type i, beta subunit |
|  |  |
| *Catecholaminergic polymorphic ventricular tachycardia (CPVT) - 5 genes* | |
| *RYR2* | ryanodine receptor 2 (cardiac) |
| *CASQ2* | calsequestrin 2 (cardiac muscle) |
| *KCNE1* | potassium voltage-gated channel, Isk-related family, member 1 |
| *KCNJ2* | potassium inwardly-rectifying channel, subfamily J, member 2 |
| *CALM1* | calmodulin 1 (phosphorylase kinase, delta) |
|  |  |
| ***Cardiomyopathies*** | |
| *Hypertrophic cardiomyopathy (HCM) - 35 genes* | |
| *MYBPC3* | myosin binding protein C, cardiac |
| *MYH7* | myosin, heavy chain 7, cardiac muscle, beta |
| *TNNT2* | troponin T type 2 (cardiac) |
| *TNNI3* | troponin I type 3 (cardiac) |
| *TPM1* | tropomyosin 1 (alpha) |
| *MYL3* | myosin, light chain 3, alkali; ventricular, skeletal, slow |
| *MYL2* | myosin, light chain 2, regulatory, cardiac, slow |
| *ACTC1* | actin, alpha, cardiac muscle 1 |
| *CSRP3* | cysteine and glycine-rich protein 3 (cardiac LIM protein) |
| *PRKAG2* | protein kinase, AMP-activated, gamma 2 non-catalytic subunit |
| *MYPN* | myopalladin |
| *TTN* | titin |
| *TCAP* | titin-cap (telethonin) |
| *TNNC1* | troponin C type 1 (slow) |
| *JPH2* | junctophilin 2 |
| *ACTN2* | actinin, alpha 2 |
| *MYH6* | myosin, heavy chain 6, cardiac muscle, alpha |
| *MYLK2* | myosin light chain kinase 2 |
| *ANKRD1* | ankyrin repeat domain 1 (cardiac muscle) |
| *VCL* | vinculin |
| *CALR3* | calreticulin 3 |
| *MYOZ2* | myozenin 2 |
| *NEXN* | nexilin (F actin binding protein) |
| *RAF1* | v-raf-1 murine leukemia viral oncogene homolog 1 |
| *CACNA1C* | calcium channel, voltage-dependent, L type, alpha 1C subunit |
| *CAV3* | caveolin 3 |
| *CASQ2* | calsequestrin 2 (cardiac muscle) |
| *DES* | desmin |
| *FXN* | frataxin |
| *GLA* | galactosidase, alpha |
| *PDLIM3* | PDZ and LIM domain 3 |
| *LAMP2* | lysosomal-associated membrane protein 2 |
| *PTPN11* | protein tyrosine phosphatase, non-receptor type 11 |
| *CRYAB* | crystallin, alpha B |
| *KCNQ1* | potassium voltage-gated channel, KQT-like subfamily, member 1 |
| *PTPN11* | protein tyrosine phosphatase, non-receptor type 11 |
| *RAF1* | v-raf-1 murine leukemia viral oncogene homolog 1 |
|  |  |
| *Dilated cardiomyopathy (DCM) - 40 genes* | |
| *LMNA* | lamin A/C |
| *MYH7* | myosin, heavy chain 7, cardiac muscle, beta |
| *TTN* | titin |
| *DSP* | desmoplakin |
| *MYBPC3* | myosin binding protein C, cardiac |
| *TNNT2* | troponin T type 2 (cardiac) |
| *SCN5A* | sodium channel, voltage-gated, type V, alpha subunit |
| *RBM20* | RNA binding motif protein 20 |
| *DMD* | dystrophin |
| *TPM1* | tropomyosin 1 (alpha) |
| *LDB3* | LIM domain binding 3 |
| *DES* | desmin |
| *TNNI3* | troponin I type 3 (cardiac) |
| *MYPN* | myopalladin |
| *MYH6* | myosin, heavy chain 6, cardiac muscle, alpha |
| *TNNC1* | troponin C type 1 (slow) |
| *ANKRD1* | ankyrin repeat domain 1 (cardiac muscle) |
| *TCAP* | titin-cap (telethonin) |
| *VCL* | vinculin |
| *PLN* | phospholamban |
| *ACTC1* | actin, alpha, cardiac muscle 1 |
| *ACTN2* | actinin, alpha 2 |
| *DSG2* | desmoglein 2 |
| *CSRP3* | cysteine and glycine-rich protein 3 (cardiac LIM protein) |
| *FKTN* | fukutin |
| *SGCD* | sarcoglycan, delta (35kDa dystrophin-associated glycoprotein) |
| *TAZ* | tafazzin |
| *CRYAB* | crystallin, alpha B |
| *NEXN* | nexilin (F actin binding protein) |
| *PKP2* | plakophilin 2 |
| *ABCC9* | ATP-binding cassette, sub-family C (CFTR/MRP), member 9 |
| *LAMA4* | laminin, alpha 4 |
| *DSC2* | desmocollin 2 |
| *TMPO* | thymopoietin |
| *LAMP2* | lysosomal-associated membrane protein 2 |
| *HFE* | HFE |
| *PDLIM3* | PDZ and LIM domain 3 |
| *JUP* | junction plakoglobin |
| *FKRP* | fukutin-related protein |
| *SDHA* | flavoprotein subunit of succinate dehydrogenase |
|  |  |
| *Arrhythmogenic right ventricular cardiomyopathy (ARVC) - 12 genes* | |
| *PKP2* | plakophilin 2 |
| *DSP* | desmoplakin |
| *DSG2* | desmoglein 2 |
| *DSC2* | desmocollin 2 |
| *JUP* | junction plakoglobin |
| *TTN* | titin |
| *TMEM43* | transmembrane protein 43 |
| *RYR2* | ryanodine receptor 2 (cardiac) |
| *DES* | desmin |
| *TGFB3* | transforming growth factor, beta 3 |
| *LMNA* | lamin A/C |
| *SCN5A* | sodium channel, voltage-gated, type V, alpha subunit |
|  |  |
| *Restrictive cardiomyopathy (RCM) - 9 genes* | |
| *TNNI3* | troponin I type 3 (cardiac) |
| *DES* | desmin |
| *MYH7* | myosin, heavy chain 7, cardiac muscle, beta |
| *TNNT2* | troponin T type 2 (cardiac) |
| *ACTC1* | actin, alpha, cardiac muscle 1 |
| *MYL3* | myosin, light chain 3, alkali; ventricular, skeletal, slow |
| *MYL2* | myosin, light chain 2, regulatory, cardiac, slow |
| *TPM1* | tropomyosin 1 (alpha) |
| *MYPN* | myopalladin |
|  |  |
| *Left ventricular non-compaction (LVNC) - 7 genes* | |
| *MYH7* | myosin, heavy chain 7, cardiac muscle, beta |
| *MYBPC3* | myosin binding protein C, cardiac |
| *TAZ* | tafazzin |
| *TPM1* | tropomyosin 1 (alpha) |
| *ACTC1* | actin, alpha, cardiac muscle 1 |
| *TNNT2* | troponin T type 2 (cardiac) |
| *CASQ2* | calsequestrin 2 (cardiac muscle) |

**ONLINE TABLE 2.** Gene level mean callability

| **Gene** | **Cohort 1** | **Cohort 2** |
| --- | --- | --- |
| *ABCC9* | 98% | 94% |
| *ACTC1* | 97% | 98% |
| *ACTN2* | 97% | 98% |
| *AKAP9* | 97% | 96% |
| *ANK2* | 98% | 97% |
| *ANKRD1* | 97% | 98% |
| *CACNA1C* | 96% | 99% |
| *CACNA2D1* | 95% | 93% |
| *CACNB2* | 97% | 98% |
| *CALM1* | 94% | 98% |
| *CALR3* | 94% | 99% |
| *CASQ2* | 98% | 98% |
| *CAV3* | 96% | 99% |
| *CRYAB* | 97% | 98% |
| *CSRP3* | 98% | 99% |
| *DES* | 95% | 99% |
| *DMD* | 96% | 93% |
| *DSC2* | 98% | 95% |
| *DSG2* | 95% | 97% |
| *DSP* | 98% | 98% |
| *FKRP* | 91% | 99% |
| *FKTN* | 92% | 95% |
| *FXN* | 91% | 99% |
| *GLA* | 82% | 98% |
| *GPD1L* | 95% | 98% |
| *HCN4* | 87% | 99% |
| *HFE* | 99% | 99% |
| *JPH2* | 94% | 98% |
| *JUP* | 95% | 99% |
| *KCNE1* | 99% | 99% |
| *KCNE2* | 99% | 98% |
| *KCNE3* | 95% | 99% |
| *KCNH2* | 91% | 99% |
| *KCNJ2* | 97% | 99% |
| *KCNJ5* | 95% | 99% |
| *KCNJ8* | 98% | 99% |
| *KCNQ1* | 88% | 99% |
| *LAMA4* | 98% | 96% |
| *LAMP2* | 92% | 95% |
| *LDB3* | 92% | 98% |
| *LMNA* | 94% | 99% |
| *MYBPC3* | 92% | 99% |
| *MYH6* | 94% | 98% |
| *MYH7* | 96% | 98% |
| *MYL2* | 97% | 99% |
| *MYL3* | 95% | 98% |
| *MYLK2* | 94% | 99% |
| *MYOZ2* | 99% | 96% |
| *MYPN* | 94% | 97% |
| *NEXN* | 92% | 96% |
| *PDLIM3* | 97% | 99% |
| *PKP2* | 96% | 98% |
| *PLN* | 97% | 98% |
| *PRKAG2* | 91% | 98% |
| *PTPN11* | 90% | 98% |
| *RAF1* | 98% | 98% |
| *RANGRF* | 95% | 98% |
| *RBM20* | 94% | 98% |
| *RYR2* | 98% | 97% |
| *SCN1B* | 77% | 97% |
| *SCN3B* | 99% | 99% |
| *SCN4B* | 95% | 99% |
| *SCN5A* | 95% | 99% |
| *SDHA* | 93% | 99% |
| *SGCD* | 99% | 97% |
| *SNTA1* | 86% | 97% |
| *TAZ* | 83% | 99% |
| *TCAP* | 88% | 99% |
| *TGFB3* | 97% | 99% |
| *TMEM43* | 94% | 99% |
| *TMPO* | 95% | 98% |
| *TNNC1* | 93% | 99% |
| *TNNI3* | 89% | 99% |
| *TNNT2* | 95% | 99% |
| *TPM1* | 97% | 99% |
| *TTN* | 93% | 95% |
| *VCL* | 98% | 98% |

**ONLINE TABLE 3.** Exons with mean callability lower than 80%

| **Chr** | **Start** | **End** | **Gene** | **Exon size (bp)** | **Mean callability** | **Cohort** |
| --- | --- | --- | --- | --- | --- | --- |
| chr7 | 81642776 | 81642826 | *CACNA2D1* | 50 | 78% | Cohort 1 |
| chrX | 31132808 | 31132816 | *DMD* | 8 | 64% | Cohort 1 |
| chr18 | 29078214 | 29078259 | *DSG2* | 45 | 68% | Cohort 1 |
| chr9 | 71650698 | 71650863 | *FXN* | 165 | 68% | Cohort 1 |
| chr15 | 73617638 | 73617785 | *HCN4* | 147 | 76% | Cohort 1 |
| chr15 | 73659826 | 73660611 | *HCN4* | 785 | 77% | Cohort 1 |
| chr7 | 150674925 | 150675001 | *KCNH2* | 76 | 74% | Cohort 1 |
| chr11 | 2466328 | 2466714 | *KCNQ1* | 386 | 58% | Cohort 1 |
| chrX | 119590505 | 119590624 | *LAMP2* | 119 | 59% | Cohort 1 |
| chr14 | 23859265 | 23859655 | *MYH6* | 390 | 79% | Cohort 1 |
| chr1 | 78389895 | 78389927 | *NEXN* | 32 | 33% | Cohort 1 |
| chr7 | 151329154 | 151329224 | *PRKAG2* | 70 | 39% | Cohort 1 |
| chr12 | 112856915 | 112856929 | *PTPN11* | 14 | 9% | Cohort 1 |
| chr12 | 112895095 | 112895130 | *PTPN11* | 35 | 70% | Cohort 1 |
| chr19 | 35521724 | 35521764 | *SCN1B* | 40 | 10% | Cohort 1 |
| chr5 | 218470 | 218533 | *SDHA* | 63 | 79% | Cohort 1 |
| chr20 | 32031116 | 32031426 | *SNTA1* | 310 | 49% | Cohort 1 |
| chrX | 153640180 | 153640289 | *TAZ* | 109 | 41% | Cohort 1 |
| chrX | 153641818 | 153641904 | *TAZ* | 86 | 76% | Cohort 1 |
| chr2 | 179519522 | 179519555 | *TTN* | 33 | 0% | Cohort 1 |
| chr2 | 179519639 | 179519722 | *TTN* | 83 | 3% | Cohort 1 |
| chr2 | 179522654 | 179522688 | *TTN* | 34 | 62% | Cohort 1 |
| chr2 | 179522999 | 179523082 | *TTN* | 83 | 6% | Cohort 1 |
| chr2 | 179523195 | 179523278 | *TTN* | 83 | 43% | Cohort 1 |
| chr2 | 179523731 | 179523815 | *TTN* | 84 | 13% | Cohort 1 |
| chr2 | 179523898 | 179523982 | *TTN* | 84 | 2% | Cohort 1 |
| chr2 | 179526675 | 179526755 | *TTN* | 80 | 20% | Cohort 1 |
| chr2 | 179527260 | 179527343 | *TTN* | 83 | 17% | Cohort 1 |
| chr2 | 179527456 | 179527539 | *TTN* | 83 | 55% | Cohort 1 |
| chr7 | 91645442 | 91645581 | *AKAP9* | 139 | 79% | Cohort 2 |
| chr2 | 179518347 | 179518428 | *TTN* | 81 | 56% | Cohort 2 |
| chr2 | 179519171 | 179519261 | *TTN* | 90 | 77% | Cohort 2 |
| chr2 | 179519471 | 179519555 | *TTN* | 84 | 54% | Cohort 2 |
| chr2 | 179519638 | 179519722 | *TTN* | 84 | 54% | Cohort 2 |
| chr2 | 179522413 | 179522494 | *TTN* | 81 | 60% | Cohort 2 |
| chr2 | 179522607 | 179522688 | *TTN* | 81 | 61% | Cohort 2 |
| chr2 | 179522998 | 179523082 | *TTN* | 84 | 60% | Cohort 2 |
| chr2 | 179523194 | 179523278 | *TTN* | 84 | 70% | Cohort 2 |
| chr2 | 179523731 | 179523815 | *TTN* | 84 | 62% | Cohort 2 |
| chr2 | 179523898 | 179523982 | *TTN* | 84 | 52% | Cohort 2 |
| chr2 | 179526674 | 179526755 | *TTN* | 81 | 65% | Cohort 2 |
| chr2 | 179526868 | 179526949 | *TTN* | 81 | 51% | Cohort 2 |
| chr2 | 179527259 | 179527343 | *TTN* | 84 | 57% | Cohort 2 |
| chr2 | 179527455 | 179527539 | *TTN* | 84 | 76% | Cohort 2 |

**ONLINE TABLE 4.** Results of rare-variants association analysis

|  | **Protein altering** | **Synonymous** | |
| --- | --- | --- | --- |
|  | **MAF1in10000 and CADD >25** | **MAF 1in1000** | **MAF 1in10000** |
| **Gene** | **Pvalue** | **Pvalue** | **Pvalue** |
| *RYR2* | 5.00E-05 | 0.890331 | 0.0620057 |
| *KCNH2* | 0.0079 | 0.502687 | 0.0591 |
| *HCN4* | 0.023 | 0.0153 | 0.196942 |
| *LDB3* | 0.0614931 | 0.496728 | 0.620295 |
| *MYPN* | 0.0924561 | 0.0339 | 0.04775 |
| *KCNQ1* | 0.0952276 | 0.436984 | 0.625825 |
| *TMEM43* | 0.120778 | 0.789 | 0.420351 |
| *TTN* | 0.157059 | 0.346995 | 0.397691 |
| *KCNJ5* | 0.164204 | 0.05735 | 0.0589345 |
| *ACTC1* | 0.168976 | 0.786 | 0.7905 |
| *SNTA1* | 0.169722 | 0.6825 | 0.6605 |
| *CALR3* | 0.17313 | 0.342769 | 0.35154 |
| *FKTN* | 0.1755 | 0.345711 | 0.6745 |
| *PLN* | 0.177683 | 0.328502 | NA |
| *SDHA* | 0.21106 | 0.263279 | 0.497835 |
| *DSG2* | 0.228935 | 0.483879 | 0.8575 |
| *JUP* | 0.272864 | 0.451196 | 0.846475 |
| *MYBPC3* | 0.274111 | 0.01075 | 0.0831663 |
| *ANK2* | 0.295426 | 0.673566 | 0.48612 |
| *CRYAB* | 0.32525 | NA | NA |
| *PDLIM3* | 0.328666 | 0.799 | 0.356012 |
| *RAF1* | 0.353028 | 0.842 | 0.788 |
| *SCN1B* | 0.395877 | 0.348797 | 0.173551 |
| *CACNB2* | 0.485201 | 0.278838 | 0.346242 |
| *AKAP9* | 0.495627 | 0.03685 | 0.75485 |
| *PKP2* | 0.495708 | 0.183743 | 0.070624 |
| *LAMA4* | 0.505061 | 0.08505 | 0.167525 |
| *NEXN* | 0.507584 | 0.4832 | 0.613365 |
| *MYH7* | 0.510116 | 0.434959 | 0.341724 |
| *ABCC9* | 0.510182 | 0.868142 | 0.716424 |
| *ACTN2* | 0.514609 | 0.615436 | 0.7875 |
| *LMNA* | 0.621763 | 0.853647 | 0.621126 |
| *SCN5A* | 0.645242 | 0.05545 | 0.0823997 |
| *FXN* | 0.666 | 0.6835 | 0.676 |
| *ANKRD1* | 0.6675 | 0.6795 | NA |
| *MYL3* | 0.668 | 0.174948 | NA |
| *HFE* | 0.669 | 0.165017 | 0.184366 |
| *TNNT2* | 0.669 | 0.7895 | 0.497842 |
| *CASQ2* | 0.6715 | 0.7895 | 0.9025 |
| *DSP* | 0.671762 | 0.248552 | 0.01075 |
| *MYOZ2* | 0.672 | 0.0602918 | 0.0611471 |
| *PRKAG2* | 0.6725 | 0.273637 | 0.62037 |
| *PTPN11* | 0.6725 | 0.0570125 | 0.336435 |
| *KCNE3* | 0.673 | NA | NA |
| *JPH2* | 0.673 | 0.687877 | 0.609823 |
| *SCN3B* | 0.673 | 0.530503 | 0.782 |
| *CACNA2D1* | 0.6745 | 0.461127 | 0.273743 |
| *MYLK2* | 0.6755 | 0.895023 | 0.780864 |
| *MYL2* | 0.6765 | 0.160823 | NA |
| *VCL* | 0.677896 | 0.123335 | 0.172951 |
| *SCN4B* | 0.684 | NA | NA |
| *CACNA1C* | 0.688 | 0.867867 | 0.711149 |
| *GPD1L* | 0.689 | 0.357904 | 0.7915 |
| *DES* | 0.7 | 0.357479 | 0.340985 |
| *MYH6* | 0.839614 | 0.647215 | 0.923489 |
| *DSC2* | 0.8715 | 0.809194 | 0.493539 |
| *RBM20* | 0.916 | 0.235256 | 0.941 |

**ONLINE REFERENCES**

1. Skinner, J. R. *et al.* Prospective, population-based long QT molecular autopsy study of postmortem negative sudden death in 1 to 40 year olds. *Heart Rhythm* **8,** 412–9 (2011).

2. Pua, C. J. *et al.* Development of a Comprehensive Sequencing Assay for Inherited Cardiac Condition Genes. *J. Cardiovasc. Transl. Res.* **9,** 3–11 (2016).
